# Choroidal microvascular proliferation secondary to diabetes mellitus

**DOI:** 10.18632/oncotarget.14020

**Published:** 2016-12-19

**Authors:** Rui Hua, Li Qing, Ian Yat Hin Wong, Hong Ning, Hailin Wang

**Affiliations:** ^1^ Department of Ophthalmology, First Hospital of China Medical University, Shenyang, China; ^2^ Department of Ophthalmology, University of Hong Kong, Hong Kong, China; ^3^ Department of Ophthalmology, The Fourth Hospital of People, Shenyang, China

**Keywords:** microangiopathy, proliferative diabetic choroidopathy, choroidal neovasculation, fluorescein angiography, indocyanine green angiography, Pathology Section

## Abstract

Diabetes is a common endocrine disorder associated with peripheral microvascular diseases such as proliferative retinal microangiopathy (or diabetic retinopathy), which may lead to blindness. Unfortunately, diabetic microvascular abnormalities in the choroid are underestimated in clinical practice. Recent literature has revealed that the severity of diabetic retinopathy is aggravated by choroidopathy resulting from hyperglycemia. Here, we introduce a case of diabetic retinopathy with choroidal neovascularization membrane but without signs of retinal microvascular proliferation or drusen. We investigated the pathogenesis of choroidal microvascular proliferation secondary to diabetes. We postulate that choroidal neovascularization is an intraocular microvascular complication of diabetes mellitus. Intravitreal anti-vascular endothelial growth factor therapy may be a treatment option for microvascular proliferation in both retina and choroids.

## INTRODUCTION

Diabetes mellitus is a common endocrine disorder associated with peripheral microvascular abnormalities such as proliferative retinal microangiopathy (or diabetic retinopathy, DR), which may lead to blindness. However, diabetic choroidal microvascular abnormalities have not received proper attention. DR and age-related macular degeneration (AMD) are two leading causes of visual impairment in older individuals in developed countries [1]. DR is generally considered to be a microvascular disease of the inner retina, in contrast to AMD involvement of the outer retina. However, there are features common to both diseases.

In the Caucasian population, diabetic patients have an increased risk of AMD compared with non-diabetic patients. In particular, patients with Type 1 and Type 2 diabetes mellitus have an approximately 2-fold and 1.4-fold increased risk of AMD, respectively [2]. Among the Asian population, in contrast, AMD is less prevalent in diabetic patients [3]. Nevertheless, an association between DR and neovascular AMD has been reported [4].

In one of our previous studies, we described diabetic choroidopathy, which was defined as the various choroidal abnormalities observed in diabetic patients, including hyperfluorescent lobules in the choriocapillaris, hypofluorescent lobules resulting from delayed filling of focal ischemic inner choriocapillaris regions, choroidal aneurysms, neovascularizations, varicose and tortuous choroidal vessels, and late phase hypoperfusion [5]. Here, we investigated the imaging characteristics of diabetic choroidopathy and the pathogenesis of choroidal microvascular proliferation secondary to diabetes mellitus.

## CASE REPORT

**Figure 1:**
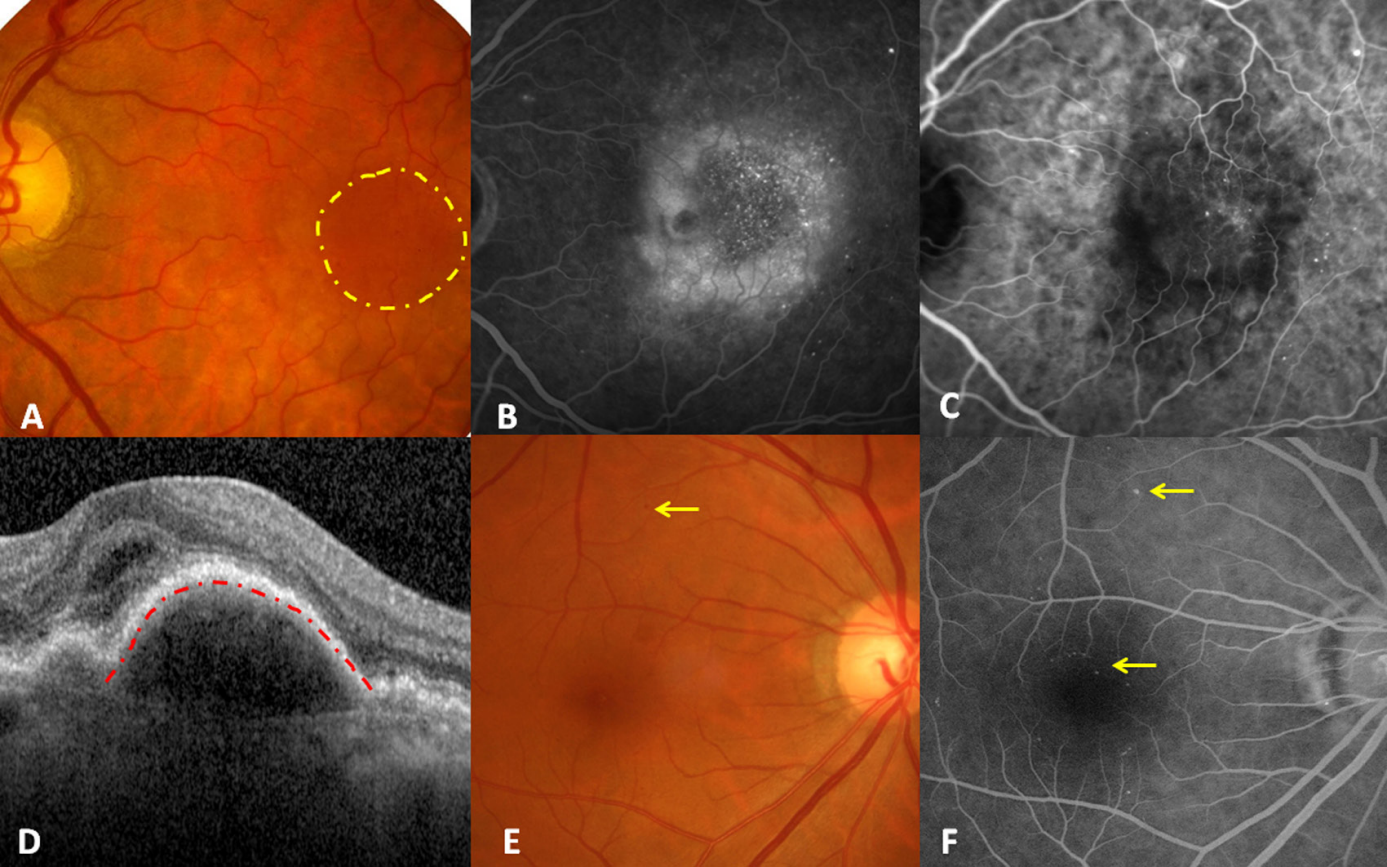
Illustration of diabetic choroidal microvascular proliferation. A. Fundus photography demonstrating sporadic retinal microaneurysms and a submacular round dark reflection involving the fovea (yellow circle), without drusen. B. Late stage of fluorescein angiography showing macular leakage, multiple hyperfluorescent dots, and the normal background fluorescence originating from RPE except for leakage. C. Late stage of indocyanine green angiography indicating multiple hyperfluorescent dots and CNV. D. Foveal horizontal optic coherence tomography showing subfoveal fluid together with RPE detachment (red line). E. Fundus photography of the right eye only demonstrated macular retinal microaneurysms. F. Late-stage fluorescein angiography of the right eye revealed more retinal microaneurysms and normal background fluorescence originating from the RPE.

Informed consent was obtained from a 48-year-old Chinese female patient with a 10-year history of type 2 diabetes, who complained of a 6-month history of blurred vision and metamorphopsia in her left eye and had no history of myopia, glaucoma, trauma or other prior interventions. Her fasting blood glucose concentration was 8.7 mmol/L and HbA1c was 8.1%. Her visual acuity was 24/60 in the left eye and 48/60 in the contralateral eye; intraocular pressure was 18 mmHg and 19 mmHg, respectively. Fundus photography of the left eye revealed sporadic retinal microaneurysms and a sub-macular round dark reflection involving the fovea, without drusen. Additionally, macular leakage, multiple hyperfluorescent dots, choroidal neovasculation (CNV) and normal background fluorescence originating from RPE except for leakage were observed in the late stage by fluorescein angiography and indocyanine green angiography, respectively. Moreover, a macular optic coherence tomography scan revealed subfoveal fluid together with detachment of the retinal pigment epithelium (RPE). According to Early Treatment Diabetic Retinopathy Study standards, these data confirmed the presence of CNV in non-proliferative DR [6]. The CNV was not secondary to AMD; hence, it was speculated that CNV formation resulted from the diabetes mellitus. Examinations of the contralateral eye also suggested the presence of non-proliferative DR (Figure 1).

## DISCUSSION

Taken together, these examinations confirmed that CNV, in this case not secondary to AMD, occurred in non-proliferative DR. The natural history of AMD is characterized by early-to-intermediate-stage disease progression followed by the development of the two major advanced forms of AMD, i.e., geographic atrophy and neovascular AMD [7]. Moreover, early AMD is associated with numerous biochemical abnormalities, including oxidative damage to RPE cells, complement deposition in the RPE-Bruch's membrane-choriocapillaris complex, lipidization of Bruch's membrane, and extracellular matrix abnormalities [8]. However, in the current study, no drusen were observed, and the RPE was normal outside the CNV in the left eye, mimicking the right eye. Hence, the authors speculated that microvascular proliferation in the choroid resulted from diabetes mellitus. We named the lesions proliferative diabetic choroidopathy (PDC).

There are several hypotheses regarding the pathogenesis of PDC. First, histopathological studies of the eyes of subjects with long-term diabetes have shown basement membrane thickening of the choriocapillaris walls, luminal narrowing, disappearance of the choriocapillaris, and thickening of Bruch's membrane, potentially predisposing the eye to the development of AMD and CNV [9]. Second, hyperglycemia and dyslipidemia in diabetic patients disturb retinal homeostasis by inducing inflammatory responses in tissue cells, including oxidative stress. Such inflammatory activation and oxidative stress lead to dysfunction and even death of RPE/photoreceptor cells [10]. Third, carbohydrate-related mechanisms have been implicated in the pathogenesis of both diseases, including the formation of advanced glycation end-products [11]. Fourth, diabetic microangiopathy due to hyperglycemia and dyslipidemia shares common pathogenic pathways with the formation of CNV in AMD. Choroidal blood flow is reduced in patients with DR. Vascular endothelial growth factor (VEGF) seems to activate both DR and CNV. Other factors, including diabetes-related apolipoproteins and mitochondrial dysfunction, contribute to the formation of AMD. Moreover, some choroidal circulatory disorders may occur prior to DR. For example, a choroidal blood flow deficit can be an early pathological change in DR [12], which may induce early choroidal microvascular proliferation. In addition, Islet-1 stimulates cell specification, differentiation, and the maintenance of phenotypes in the vertebrate neural retina [13].

Reduction of the VEGF concentration using anti-VEGF drugs appears to be a valuable treatment option for patients with neovascular AMD and concomitant DR. Intravitreal anti-VEGF therapy for eyes with both neovascular AMD and DR stabilizes vision and reduces central macula thickness [14]. Anti-VEGF halts the progression of DR and even reverses the grade of DR. The common pathological microvascular features of these diseases may be influenced by anti-VEGF. Therefore, the presence of elevated VEGF levels and the secondary underlying changes in the choriocapillaris in DR may alter the disease course and treatment response for CNV.

In conclusion, we postulate that CNV formation might be an ocular microvascular complication of diabetes mellitus. This condition was detected prior to retinal neovascularization of DR in our patient, indicating earlier diabetic choroidopathy. Early investigation of choroidal microvascular proliferation in diabetic patients with visual symptoms may be warranted. Furthermore, anti-VEGF may be a potential treatment option for both diabetic retinopathy and choroidopathy.

## References

[1] Congdon NG, Friedman DS, Lietman T Important causes of visual impairment in the world today. JAMA.

[2] Vassilev ZP, Ruigomez A, Soriano-Gabarro M, Garcia Rodriguez LA Diabetes, cardiovascular morbidity, and risk of age-related macular degeneration in a primary care population. Invest Ophthalmol Vis Sci.

[3] Cho BJ, Heo JW, Shin JP, Ahn J, Kim TW, Chung H Epidemiological association between systemic diseases and age-related macular degeneration: the Korea National Health and Nutrition Examination Survey 2008-2011. Invest Ophthalmol Vis Sci.

[4] Topouzis F, Anastasopoulos E, Augood C, Bentham GC, Chakravarthy U, de Jong PT, Rahu M, Seland J, Soubrane G, Tomazzoli L, Vingerling JR, Vioque J, Young IS, Fletcher AE Association of diabetes with age-related macular degeneration in the EUREYE study. Br J Ophthalmol.

[5] Hua R, Liu L, Wang X, Chen L Imaging evidence of diabetic choroidopathy in vivo: angiographic pathoanatomy and choroidal-enhanced depth imaging. PLoS One.

[6] Early Treatment Diabetic Retinopathy Study Research Group. Grading diabetic retinopathy from stereoscopic color fundus photographs--an extension of the modified Airlie House classification. ETDRS report number 10. Ophthalmology.

[7] Holz FG, Schmitz-Valckenberg S, Fleckenstein M Recent developments in the treatment of age-related macular degeneration.J. Clin Invest.

[8] Zarbin MA, Casaroli-Marano RP, Rosenfeld PJ Age-related macular degeneration: clinical findings, histopathology and imaging techniques.Dev. Ophthalmol.

[9] Hidayat AA Fine BS.Diabetic choroidopathy. Light and electron microscopic observations of seven cases. Ophthalmology.

[10] Zhang W, Liu H, Al-Shabrawey M, Caldwell RW, Caldwell RB Inflammation and diabetic retinal microvascular complications. J Cardiovasc Dis Res.

[11] Howes KA, Liu Y, Dunaief JL, Milam A, Frederick JM, Marks A, Baehr W Receptor for advanced glycation end products and age-related macular degeneration. Invest Ophthalmol Vis Sci.

[12] Muir ER, Rentería RC, Duong TQ Reduced ocular blood flow as an early indicator of diabetic retinopathy in a mouse model of diabetes. Invest Ophthalmol Vis Sci.

[13] Martín-Partido G, Francisco-Morcillo J The role of Islet-1 in cell specification, differentiation, and maintenance of phenotypes in the vertebrate neural retina.Neural. Regen Res.

[14] Bandello F, Corvi F, C La Spina, Benatti L, Querques L, Capuano V, Naysan J, Chen X, Sarraf D, Parodi MB, Souied E, Freund KB, Querques G Outcomes of intravitreal anti-VEGF therapy in eyes with both neovascular age-related macular degeneration and diabetic retinopathy. Br J Ophthalmol.

